# Application of Silibinin Oleate as a Nutraceutical Antioxidant for Improving the Quality of Sunflower Oil

**DOI:** 10.3390/molecules31071222

**Published:** 2026-04-07

**Authors:** Cristina Adriana Dehelean, Cristian Oancea, Andreea-Adriana Neamtu, Vlad Enache, Victor Emil Alexa, Ileana Cocan, Mariana Suba, Maria-Alexandra Pricop, Alexandra Teodora Lukinich-Gruia, Călin Adrian Tatu, Ersilia Alexa

**Affiliations:** 1 Faculty of Pharmacy, “Victor Babes” University of Medicine and Pharmacy Timisoara, Eftimie Murgu Square, No. 2, 300041 Timisoara, Romania; cadehelean@umft.ro (C.A.D.); andreea.neamtu@umft.ro (A.-A.N.); 2 Research Center for Pharmaco-Toxicological Evaluations, Faculty of Pharmacy, “Victor Babes” University of Medicine and Pharmacy Timisoara, Eftimie Murgu Square, No. 2, 300041 Timisoara, Romania; 3 Doctoral School, University of Life Sciences “King Mihai I” from Timisoara, Aradului Street No. 119, 300645 Timisoara, Romania; vlad.enache@usvt.ro; 4 Faculty of Medicine, “Victor Babes” University of Medicine and Pharmacy Timisoara, Eftimie Murgu Square, No. 2, 300041 Timisoara, Romania; oancea@umft.ro (C.O.); victor-emil.alexa@student.umft.ro (V.E.A.); 5 Faculty of Food Engineering, University of Life Sciences “King Mihai I” from Timisoara, Aradului Street No. 119, 300645 Timisoara, Romania; ileanacocan@usvt.ro; 6 “Food Science” Research Center, University of Life Sciences “King Mihai I” from Timisoara, Aradului Street No. 119, 300645 Timisoara, Romania; 7 Romanian Academy, “Coriolan Dragulescu” Institute of Chemistry, Mihai Viteazu No. 24, 300223 Timisoara, Romania; marianasuba@gmail.com; 8 OncoGen Centre, County Hospital Pius Branzeu, 156 Liviu Rebreanu Boulevard, 300736 Timisoara, Romania; alexandra.pricop@oncogen.ro (M.-A.P.); alexandra.gruia@hosptm.ro (A.T.L.-G.); tatu.calin@umft.ro (C.A.T.); 9 Department of Applied Chemistry and Environmental Engineering and Inorganic Compounds, Faculty of Industrial Chemistry, Biotechnology and Environmental Engineering, Politehnica University Timisoara, Vasile Pârvan 6, 300223 Timisoara, Romania; 10 Department of Functional Sciences, Center of Immuno-Physiology (CIFBIOTEH), “Victor Babes” University of Medicine and Pharmacy Timisoara, Eftimie Murgu Square, No. 2, 300041 Timisoara, Romania

**Keywords:** peroxide value (PV), p-anisidine value (p-AV), total oxidation value (TOTOX), fatty acids, FTIR

## Abstract

Sunflower oil is particularly prone to thermo-oxidative degradation due to its high content of polyunsaturated fatty acids, especially under high-temperature conditions. This study investigated the oxidative stability of sunflower oil heated at 180 °C for 4 and 8 h, focusing on the protective effect of silibinin oleate (SIL-O), a lipophilic polyphenolic derivative, compared to the synthetic antioxidant butylated hydroxytoluene (BHT). Oxidative changes were evaluated through peroxide value (PV), p-anisidine value (p-AV), and total oxidation value (TOTOX), while structural alterations were monitored using FTIR spectroscopy. Additionally, fatty acid composition was analyzed by GC-MS to assess compositional changes associated with oxidation. Thermal treatment led to increases in PV, p-AV, and TOTOX, indicating progressive oxidation, alongside a decrease in unsaturated fatty acids. FTIR analysis revealed characteristic changes, including a reduction in the unsaturation band (~3008 cm^−1^), modifications in the ester carbonyl region (~1743 cm^−1^), and the emergence of bands associated with cis–trans isomerization (~968–970 cm^−1^). Strong correlations were observed between fatty acid degradation, FTIR indices, and oxidation parameters. Compared to the control, SIL-O inhibited oxidation in a dose-dependent manner. At 300 ppm, it outperformed BHT, demonstrating its potential as a natural antioxidant for enhancing the stability of sunflower oil during high-temperature processing.

## 1. Introduction

Sunflower oil is one of the most widely used vegetable oils due to its availability, nutritional profile and wide applicability in cooking and processing. However, in particular, the “linoleic” variants (rich in polyunsaturated fatty acids) show a high susceptibility to oxidation, especially under prolonged storage conditions, exposure to light/oxygen and, critically, to thermal treatments such as frying [1]. Lipid oxidation leads to the degradation of triacylglycerols and the formation of primary (hydroperoxides) and secondary products (aldehydes, ketones, etc.), responsible for rancidity, loss of sensory quality, decreased nutritional value and the generation of potentially undesirable compounds for health. In the recent literature, refined sunflower oil is reported to be among the most prone to oxidative degradation during frying, with pronounced increases in the peroxide value (PV), the anisidine index (p-AnV) and the global TOTOX index [1,2,3].

In this context, antioxidant supplementation remains one of the most effective strategies to delay the initiation and propagation of radical reactions in the lipid matrix. Traditionally, synthetic antioxidants (such as BHT, BHA, TBHQ, etc.) have been used, but interest in natural alternatives has increased significantly, both due to consumer demand for “clean labels” and due to recurring discussions regarding chronic exposure and long-term safety of some synthetic additives, even if they are regulated and used within the permitted limits [4,5,6].

Several studies have demonstrated the effectiveness of phenolic compounds as natural antioxidants in edible oils. Phenolic acids, flavonoids, and related derivatives act as radical scavengers through hydrogen atom transfer and electron transfer mechanisms, significantly delaying lipid oxidation [7,8,9]. A positive correlation between total phenolic content and oxidative stability has been reported in various oils, including rapeseed and commercial vegetable oils [7]. Moreover, the addition of natural phenolics such as thymol, carvacrol, or plant extracts to sunflower oil has been shown to reduce peroxide and p-anisidine values, improving thermal and storage stability [7]. These findings highlight the growing interest in using phenolic-based additives as alternatives to synthetic antioxidants in lipid systems.

A major obstacle in the utilization of many natural antioxidants (especially polyphenols) in oils is their polarity: hydrophilic compounds have low solubility in the lipid phase and may have limited efficiency in lipid-rich systems. As a solution, lipophilization of antioxidant molecules by esterification with fatty acids has become an important direction, as it increases the affinity for the lipid matrix and for the lipid–oxygen interface, where oxidation processes take place [10,11].

In this line, silibinin also falls, the main bioactive constituent of silymarin from *Silybum marianum* (milk thistle), recognized for its antioxidant properties, but limited by stability/solubility in certain matrices [12,13,14].

Silibinin oleate (SIL-O) is a lipophilic derivative of silibinin obtained by conjugation with oleic acid, proposed initially to optimize its physicochemical properties. SIL-O can be classified as silibinin derivative-based nutraceuticals, as they originate from a natural bioactive compound and are structurally modified to enhance lipophilicity, stability, and functional antioxidant performance in lipid systems. Its biocompatibility profile indicates a better tolerability for SIL-O compared to a linoleic analogue in *in vitro*/*in ovo* assays (dose-dependent) [7]. From a food perspective, this lipophilization is relevant because a more lipid-phase compatible antioxidant can act more efficiently on peroxyl radicals and delay the accumulation of both primary and secondary oxidation products [15,16,17].

Recent evidence supports the potential of lipophilic derivatives of silibinin in edible oils: a silibinin-linoleate derivative significantly reduced PV, p-AnV and TOTOX in sunflower oil subjected to heating at 180 °C (dose-dependent effect), and at the maximum concentration tested showed comparable or even superior efficiency to BHT for some parameters [3].

These results indicate that “anchoring” silibinin with an acyl chain can enhance antioxidant performance in lipid systems, but data specifically dedicated to SIL-O as a natural antioxidant for sunflower oil are still limited.

The present study aims to evaluate the potential of silibinin oleate (SIL-O) as a natural antioxidant for stabilizing PUFA-rich oils. Specifically, the objectives are to: (i) enhance the oxidative stability of sunflower oil under thermal stress and accelerated storage conditions, (ii) preserve key physicochemical properties, including fatty acid composition, oxidation indices, and frying performance, and (iii) contribute to the development of natural, functional alternatives to synthetic antioxidants, in line with current strategies for improving lipid stability, such as the use of plant-derived extracts, oil blending, and bioactive compounds.

Therefore, the study contributes in an original way to the field of natural lipophilic antioxidants and provides new data, relevant for the development of sustainable solutions for oxidative stabilization of vegetable oils used in high-temperature applications.

The choice of sunflower oil was motivated by its widespread use in frying applications and its pronounced susceptibility to thermal oxidation, providing a sensitive model for evaluating the antioxidant performance of SIL-O.

## 2. Results and Discussion

### 2.1. Evolution of Primary Oxidation (PV), Secondary Oxidation (p-AV) and Integrated Oxidation Assessment (TOTOX)

Table 1 presents Peroxide value (PV), p-Anisidine value (p-AV) and TOTOX value for oil samples with BHT and SIL-O additives subjected to heat treatment.

Peroxide value (PV) is an indicator of primary oxidation, reflecting the formation of hydroperoxides in the early stages of lipid degradation. p-Anisidine value (p-AV) evaluates secondary oxidation, by quantifying aldehydes formed from the decomposition of hydroperoxides and TOTOX provides an integrated assessment of the oxidative state capturing the transition from incipient oxidation to advanced degradation and being essential for assessing the overall oxidative stability of oils, especially under thermal processing conditions.

At T0, all samples have low PVs (≈0.96–1.75 meq O_2_/kg), indicating a relatively fresh initial oil and a low hydroperoxide loading. This range is consistent with values reported for unheated refined oils [18]; however, compared to data obtained under similar thermal conditions, the evolution of PV in the present study highlights the sensitivity of sunflower oil to thermo-oxidative stress. While PV is widely recognized as an indicator of primary oxidation, the rate and extent of its increase observed here suggest that the applied conditions promote rapid hydroperoxide formation, providing a suitable framework to evaluate the effectiveness of antioxidant treatments.

After 4 h (T4), PV increases strongly in all variants, most pronounced in Control (15.93 meq O_2_/kg). This increase is due to the formation of hydroperoxides in the initial stage of thermo-oxidation, a phenomenon consistently described for PUFA-rich oils (such as sunflower) subjected to ~180 °C [18,19]. In comparison, antioxidants reduce PV at T4: BHT (11.75) and SIL-O (11.49; 10.63; 9.78 for 100/200/300 ppm), indicating inhibition of primary product formation.

In sunflower oil heated to 180 °C, the peroxide value (PV) typically increases during the initial stages due to hydroperoxide formation, followed by a decline at prolonged heating times as these primary oxidation products decompose into secondary compounds such as aldehydes and ketones [20,21]. In the present study, this behavior provides a useful framework for interpreting the transition between primary and secondary oxidation processes and for assessing the effectiveness of antioxidant treatments under thermal stress. After 8 h (T8), PV reaches maximum values in Control (32.03 meq O_2_/kg), which signals advanced oxidation under severe heating conditions. This behavior is consistent with observations reported under frying conditions [22,23]. However, the evolution of PV is not solely indicative of oxidation intensity, but rather reflects the dynamic balance between hydroperoxide formation and their subsequent decomposition. In this context, the trends observed in the present study highlight the importance of considering both processes simultaneously when evaluating oxidative stability under thermal stress [22,23]. At this stage, SIL-3 (300 ppm) remains the variant with the minimum PV (19.48), and BHT and SIL-2 are intermediate (≈22.9–23.0), suggesting a more robust protection at the higher dose of SIL-O.

The index of secondary oxidation (p-AV) at T0 are moderate and similar between samples (≈3.89–4.41), indicating a low initial loading in secondary aldehydes. After heating, p-AV increases substantially at T4, confirming the shift from primary oxidation to the accumulation of more stable by-products (mainly aldehydes), as described in the reference studies on heated sunflower oil [18,19].

At T4, Control shows high p-AV (24.71), and BHT and SIL-3 have lower values (19.97 and 18.85), indicating reduced by-product formation. In contrast, SIL-2 has higher p-AV (32.02), a result that may suggest (i) the decomposition of hydroperoxides to aldehydes at this dose or a faster transition to secondary oxidation in a system where PV is still reduced compared to Control. The literature indicates that peroxide value (PV) and p-anisidine value (p-AV) do not necessarily evolve in parallel during lipid oxidation. PV may initially increase and subsequently stabilize or even decrease as primary oxidation products decompose, while p-AV continues to rise due to the accumulation of secondary oxidation compounds. Therefore, a correct assessment of oxidative status requires an integrated interpretation of PV and p-AV, complemented by the TOTOX index, which provides a more comprehensive evaluation of lipid oxidation [23].

At T8, p-AV increases in all samples, but remains lower in the antioxidant variants, especially SIL-3 (27.37) and BHT (29.31), compared to Control (36.06). This trend aligns with previous findings indicating that antioxidants can influence multiple stages of lipid oxidation, not only by reducing hydroperoxide formation but also by limiting the subsequent generation of secondary oxidation products such as aldehydes during thermal processing [18,19].

At 180 °C, after 8 h, some studies report PVs in the range of ~20–50 meq O_2_/kg for oils without antioxidants or with insufficient protection (the exact value depends heavily on the heating and refreshing/feeding regime) [21,24].

The TOTOX value synthesizes the impact of primary (PV) and secondary (p-AV) oxidation and is used in the oil heating literature as a global indicator of oxidative deterioration. In the analyzed set, TOTOX increases from ≈5.81–7.85 (T0) to ≈38.40–56.58 (T4) and reaches ≈73.26–93.37 (T8) in most samples, confirming the acceleration of oxidation with the extension of heating [25].

Compared between treatments, Control consistently has the highest TOTOX values (56.58 at T4; 93.37 at T8), reflecting the most severe degradation. BHT reduces TOTOX at T4 (43.47) and remains lower than Control at T8 (82.16), confirming the antioxidant effect, in agreement with studies using BHT when heating sunflower oil.

Among the SIL-O samples, SIL-3 presents the best overall profile: TOTOX is minimal at T4 (38.40) and remains very low at T8 (38.96), confirming the strong inhibition of both primary oxidation and by-product accumulation. Such behavior is consistent with recent literature investigating silibinin esters (lipophilic derivatives) as antioxidants in oils subjected to heating at 180 °C (4–8 h) [3].

Overall, heating to 180 °C causes a major increase in PV, p-AV and TOTOX, demonstrating the progression from primary oxidation to severe secondary oxidation. Compared to Control, both BHT (200 ppm) and SIL-O reduce oxidation, but SIL-O at 300 ppm provides the best overall protection (lower PV and p-AV and minimal TOTOX), supporting the potential of lipophilic silibinin derivatives as effective antioxidants in vegetable oils subjected to heat stress.

### 2.2. Changes of ATR-FTIR Spectrum

Based on the FTIR spectra at T0, T4 (4 h/180 °C) and T8 (8 h/180 °C) for sunflower oil (Control), respectively, oil added with BHT 200 ppm and SIL-O 100/200/300 ppm (SIL-1/2/3), a typical picture of thermo-oxidative oxidation of triglycerides is observed, with clear differences between treatments.

**Figure 1:**
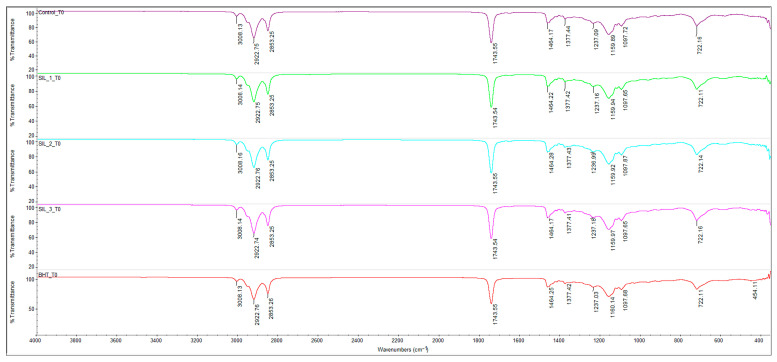
FTIR spectra of oil samples without heating: CO-control sample, sunflower oil (purple line), SIL_1_T0 sunflower oil with 100 ppm SIL-O (green line), SIL_2_T0 sunflower oil with 200 ppm SIL-O (blue line), SIL_3_T0 sunflower oil with 300 ppm SIL-O (pink line) and BHT_T0 sunflower oil with 200 ppm BHT (red line). Spectral range of 4000–400 cm^−1^, 32 scans at 4 cm^−1^ resolution.

At T0 (Figure 1), all samples show the characteristic bands of triglycerides: (a) 3008 cm^−1^ (=C–H stretch, specific to unsaturated fatty acids), (b) 2922 and 2853 cm^−1^ (C–H stretches of aliphatic chains), (c) 1743 cm^−1^ (C=O ester–carbonyl group of triglycerides), (d) 1464/1377 cm^−1^ (CH_2_/CH_3_ deformations), (e) 1237–1097 cm^−1^ (C–O ester vibrations), (f) 722 cm^−1^ ((CH_2_)_n_ “rocking”, associated with the structure of the chains).

The differences between Control, BHT and SIL at T0 are minor, suggesting that additivity (especially at ppm doses) does not change the overall FTIR “fingerprint” of triglycerides, but rather the evolution under thermal stress.

**Figure 2:**
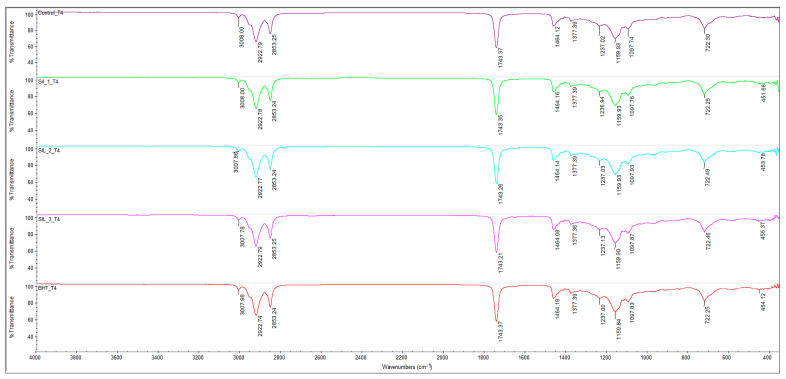
FTIR spectra of oil samples at T4 (after 4 h of heating at 180 °C): CO_T4-control sample, sunflower oil (purple line), SIL_1_T4 sunflower oil with 100 ppm (green line), SIL_2_T4 sunflower oil with 200 ppm SIL-O (blue line), SIL_3_T4 sunflower oil with 300 ppm SIL-O (pink line) and BHT_T4 sunflower oil with 200 ppm BHT (red line). Spectral range of 4000–400 cm^−1^, 32 scans at 4 cm^−1^ resolution.

Heating the oil to 180 °C caused spectral changes characteristic of thermo-oxidative degradation of triglycerides (Figure 2). A progressive diminution of the band located around ~3008 cm^−1^, associated with the =C–H stretching vibrations of unsaturated fatty acids, indicates the consumption of double bonds during the heat treatment. At the same time, the ester carbonyl region (~1743 cm^−1^) exhibited variations in band intensity (Table 1) and shape, which may be associated with overall changes in carbonyl-containing compounds within the system during thermal oxidation. Given the known overlap between triglyceride ester bands and those of oxidation products, these modifications are interpreted as indicative of alterations in the lipid matrix and the progressive formation of secondary oxidation compounds, rather than being assigned to specific molecular species.

**Figure 3:**
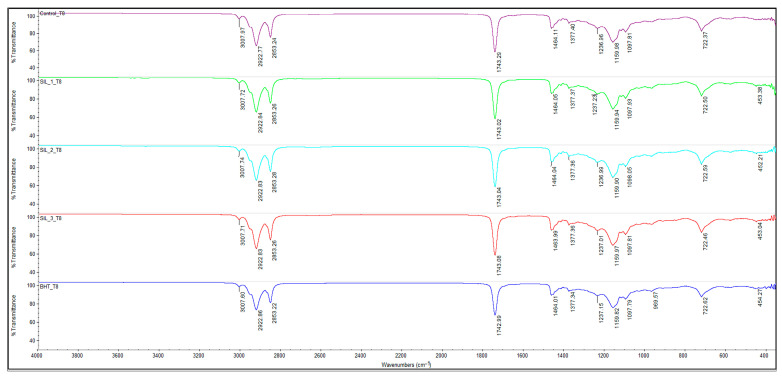
FTIR spectra of oil samples at T8 (after 8 h of heating at 180 °C): CO_T8-control sample, sunflower oil (purple line), SIL_1_T8 sunflower oil with 100 ppm SIL-O (green line), SIL_2_T8 sunflower oil with 200 ppm SIL-O (blue line), SIL_3_T8 sunflower oil with 300 ppm SIL-O (pink line) and BHT_T8 sunflower oil with 200 ppm BHT (red line). Spectral range of 4000–400 cm^−1^, 32 scans at 4 cm^−1^ resolution.

In addition, at advanced heating times (T8) (Figure 3), the appearance and intensification of a band around ~968–970 cm^−1^ was observed, frequently attributed to cis → trans isomerization processes and/or the formation of conjugated structures in the fatty acid chains. The clear presence of this signal, particularly highlighted in sample BHT_T8, confirms the progression of structural degradation of the oil under the action of high temperatures.

The comparison of treatments highlights clear differences in the evolution of thermo-oxidative degradation of sunflower oil, reflected by the FTIR spectral changes depending on the type and efficiency of the antioxidant used. The Control sample (untreated oil) shows the most pronounced changes as the heating time progresses, characterized by a sharp decrease in the band associated with unsaturation (~3008 cm^−1^), significant perturbations in the carbonyl region (~1743 cm^−1^) and an intensification of the band around ~968–970 cm^−1^, attributed to cis → trans isomerization and the formation of conjugated structures. This behavior is characteristic of PUFA-rich oils such as sunflower oil; however, beyond confirming known oxidation patterns, it reflects the high susceptibility of these systems to advanced thermo-oxidative degradation, making them particularly suitable models for evaluating antioxidant performance under frying conditions [26,27].

It should be noted that, although SIL-O may undergo thermal transformations under these conditions, no distinct FTIR bands could be unambiguously assigned to specific degradation products of the antioxidant. This is mainly due to its relatively low concentration (maximum 300 ppm) compared to the lipid matrix, leading to spectral masking by the dominant triglyceride signals. Moreover, the FTIR technique applied in this study provides a global fingerprint of the system and is not sufficiently selective to track the degradation profile of minor components within complex oil matrices. Therefore, the interpretation of the spectra was focused on matrix-level changes associated with lipid oxidation, while the antioxidant performance of SIL-O was inferred indirectly through its ability to modulate these transformations, as supported by complementary chemical and compositional analyses.

Treatment with BHT (200 ppm) leads to an attenuation of these changes compared to the control sample, indicating a clear antioxidant effect in the early stages of heating. However, at longer times (T8), the FTIR spectra of the BHT sample still show clear markers of oxidative degradation, including the distinct appearance of the ~969 cm^−1^ band, suggesting that, under severe heat treatment conditions, the efficiency of BHT is limited. This result is consistent with data in the literature, which show that synthetic antioxidants can delay oxidation, but cannot completely prevent structural degradation of lipids at high temperatures and long exposures [28,29].

In contrast, samples added with silibinin oleate (SIL-1, SIL-2, SIL-3) show a better preservation of the spectral characteristics associated with native triglycerides, especially by maintaining a relatively higher unsaturation band (~3008 cm^−1^) and by limiting the changes in the carbonyl region (~1743 cm^−1^).

The comparative analysis of antioxidant treatments indicates that the efficiency of SIL-O is strongly concentration-dependent. At intermediate levels (200 ppm), the behavior of SIL-O is comparable to that of BHT, suggesting a similar ability to limit thermo-oxidative degradation under equivalent conditions. However, at higher concentration (300 ppm), SIL-O provides improved preservation of the lipid matrix, as reflected by the FTIR-derived parameters, indicating an enhanced antioxidant effect.

These results suggest that SIL-O does not inherently outperform BHT at equal concentrations, but rather exhibits a dose-dependent increase in effectiveness, becoming more efficient at higher levels. This behavior is consistent with the mechanism of lipophilic antioxidants, where increased concentration improves localization and radical scavenging efficiency within the lipid phase.

These observations are supported by recent studies reporting that lipophilic derivatives of polyphenols, such as esterified silibinin, may exhibit superior antioxidant efficiency in lipid matrices, due to their increased solubility in the lipid phase and the ability to intercept free radicals in the vicinity of fatty acid double bonds [29,30].

The efficacy of SIL-O in heated oils results from the combination of an antioxidant polyphenolic core (silibinin) with a lipophilic chain (oleate), which increases affinity for the lipid phase and positioning in areas where oxidation is initiated and propagated (in the vicinity of fatty acid double bonds). This “lipophilization” is a recognized strategy for enhancing the antioxidant activity of flavonoids in lipid environments, by increasing solubility and access to radicals formed in the fat matrix [31,32,33].

Silibinin oleate (SIL-O) acts as a chain-breaking antioxidant by intercepting lipid-derived radicals generated during thermal and oxidative degradation of unsaturated fatty acids. During heating (180 °C), PUFA-rich oils generate lipid (L•) and peroxyl (LOO•) radicals, and oxidation proceeds in a chain manner. Silibinin (the antioxidant component of SIL-O) can act as a hydrogen/electron donor, reducing LOO• radicals and interrupting the propagation of the reaction, with the formation of a more stable phenoxyl radical (resonance), which reduces the formation of hydroperoxides and by-products (aldehydes, ketones). This mechanism is frequently described for polyphenols/flavonoids and is invoked in reviews on dietary antioxidants and inhibition of lipid peroxidation [9,33]. At the spectral level, the inhibition of degradation is reflected by: relative maintenance of the ~3008 cm^−1^ band (=C–H, unsaturation), limitation of perturbations in the carbonyl region ~1743 cm^−1^ (carbonyl oxidized products), reduction of the increase of the ~968–970 cm^−1^ band (cis → trans isomerization/conjugated structures) [34]. This pattern has been associated with improved preservation of triglyceride structures and reduced formation of oxidation products in FTIR-based studies of heated oils, supporting its use as an indicator of antioxidant effectiveness under thermal conditions [35].

Under severe regimes (180 °C, hours), antioxidants can be consumed/transformed. In the recent literature for silibinin esters in oils, the protective effect is reported to be concentration-dependent, and at higher doses it can become comparable to reference antioxidants (e.g., BHT), which is consistent with the observation that at T8 more defined differences between treatments appear [3].

In order to evaluate the effect of natural antioxidants on sunflower oil oxidative protection, the FTIR unsaturation index (A3007/A2922) for all samples was calculated (Table 2).

The FTIR unsaturation index A3007/A2922 reflects the ratio between the =C–H vibrations associated with cis double bonds (~3007 cm^−1^) and the C–H vibrations of saturated aliphatic chains (~2922 cm^−1^), being used as a relative marker of the degree of unsaturation of oils. The evolution of this index provides direct information on the stability of double bonds during heat treatment.

At the initial time (T0), all samples show very close values of the A3007/A2922 ratio (1.473–1.475), regardless of the treatment (Control, BHT or SIL-1/2/3). This uniformity confirms that the addition of SIL-O or BHT does not modify the initial structure of the lipid fraction and that all samples start from a comparable level of unsaturation, typical of sunflower oil rich in polyunsaturated fatty acids.

After 4 h of heating at 180 °C (T4), a slight increase in the A3007/A2922 ratio is observed for all samples (1.479–1.485). This apparently paradoxical increase in the unsaturation index has also been reported in the FTIR literature and is attributed to: (i) the temporary reorganization of the molecular environment of the triglycerides, (ii) the possible formation of conjugated systems or changes in the relative absorption of the FTIR bands, (iii) the fact that the oxidation is still dominated by the formation of hydroperoxides, without the massive destruction of double bonds.

The slightly higher values for the samples with SIL-O, especially SIL-3_T4 (1.485), suggest an early stabilization of the double bonds, compatible with the chain-breaking antioxidant action of the silibinin derivative [36].

After 8 h of heating (T8), the behavior of the samples clearly differs. The Control_T8 sample shows a dramatic decrease in the A3007/A2922 ratio to 0.674, indicating extensive consumption of the double bonds by oxidation, cis → trans isomerization and advanced thermal degradation.

A similar behavior is observed for the samples added with SIL-O (SIL-1_T8, SIL-2_T8, SIL-3_T8), which show close values (0.671–0.672). This result indicates that, under severe and prolonged thermal stress conditions (180 °C, 8 h), the structural degradation of the unsaturated fraction becomes dominant, and the FTIR unsaturation index reflects the global loss of double bonds, even in the presence of the natural antioxidant. In contrast, the BHT_T8 sample presents a significantly higher value of the A3007/A2922 ratio (1.350), suggesting a better preservation of the signal associated with unsaturation. This behavior can be explained by: (i) the relatively higher thermal stability of BHT, (ii) differences in the mode of interaction with the lipid structure, (iii) or possible indirect spectral interferences that maintain the relative intensity of the ~3007 cm^−1^ band [36,37,38].

The sharp decrease in the A3007/A2922 ratio at T8 for Control and SIL-O samples is in full agreement with increased values of PV, p-AV and TOTOX, confirming the progression towards advanced secondary oxidation and structural degradation of unsaturated fatty acids. FTIR thus confirms, at the molecular level, the results obtained by classical chemical methods.

Overall, the FTIR unsaturation index highlights that SIL-O contributes to the stabilization of the unsaturated fraction in the initial phases of heating (T4), but at prolonged thermal exposures (T8) the structural degradation of double bonds becomes dominant. This result underlines the importance of correlating FTIR with chemical oxidation indicators and confirms that FTIR is a complementary, not a substitute, method for assessing the overall oxidative stability of oils.

### 2.3. Evolution of Main Fatty Acid Classes

**Figure 4:**
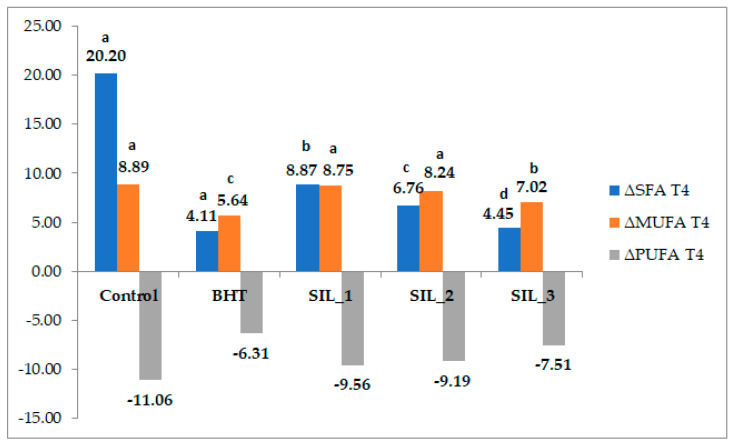
The evolution of fatty acid composition during thermal treatment assessed by analyzing the relative changes in the main fatty acid classes (SFA, MUFA, and PUFA) at T4 compared to the initial state (T0). Data for each group of fatty acids class with different superscript letters are significantly different (*p* < 0.05).

**Figure 5:**
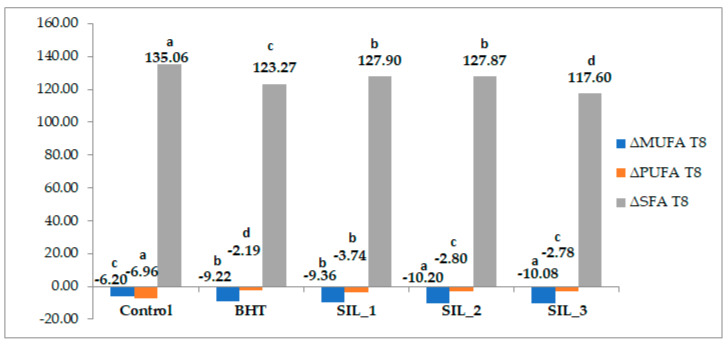
The evolution of fatty acid composition during thermal treatment assessed by analyzing the relative changes in the main fatty acid classes (SFA, MUFA, and PUFA) at T8 compared to the initial state (T0). Data for each group of fatty acids class with different superscript letters are significantly different (*p* < 0.05).

The evolution of fatty acid composition during thermal treatment was assessed by analyzing the relative changes in the main fatty acid classes (SFA, MUFA, and PUFA) at T4 and T8 compared to the initial state (T0). (Figure 4 and Figure 5).

At T4, all samples exhibited a negative ΔPUFA (−7.11% to −11.06%), accompanied by a positive ΔSFA (+4.45% to +20.20%) and positive ΔMUFA values. This behavior is consistent with the well-documented mechanism of lipid oxidation in PUFA-rich oils, where polyunsaturated fatty acids are preferentially oxidized due to the higher reactivity of their multiple double bonds. The decrease in PUFA and the concomitant increase in the relative proportion of SFA and MUFA reflect an early stage of thermo-oxidative degradation, frequently reported in sunflower oil subjected to frying conditions [39].

The control sample showed the most pronounced PUFA loss (ΔPUFA = −11.06%) and the highest increase in SFA (ΔSFA = +20.20%), indicating rapid oxidation in the absence of antioxidants. In contrast, samples containing antioxidants exhibited attenuated changes, confirming their protective effect. Among them, BHT (ΔPUFA = −7.11%) and SIL-3 (ΔPUFA = −7.51%) demonstrated the highest efficiency in limiting early-stage oxidation, while SIL-1 and SIL-2 showed intermediate behavior. These findings are in agreement with previous studies demonstrating that antioxidants can effectively delay the formation of hydroperoxides and reduce the rate of PUFA degradation under thermal stress [39]. PUFA-rich oils are typically characterized by high unsaturation index values, which are directly associated with their increased susceptibility to oxidative degradation, particularly under thermal stress conditions [21,26]. At T8, the oxidation process progressed to a more advanced stage, as indicated by the substantial increase in ΔSFA (+117–135%) and the transition of ΔMUFA to negative values (−6.20% to −10.20%), suggesting that monounsaturated fatty acids also became affected by oxidative reactions. This shift reflects the transition from primary to secondary oxidation processes, where not only PUFA but also more stable lipid fractions undergo degradation [40].

The observed trends are consistent with the general understanding that lipid oxidation in sunflower oil is driven primarily by the degradation of polyunsaturated fatty acids, and that antioxidant addition can significantly modulate this process.

### 2.4. The Changes in Color Parameters

**Figure 6:**
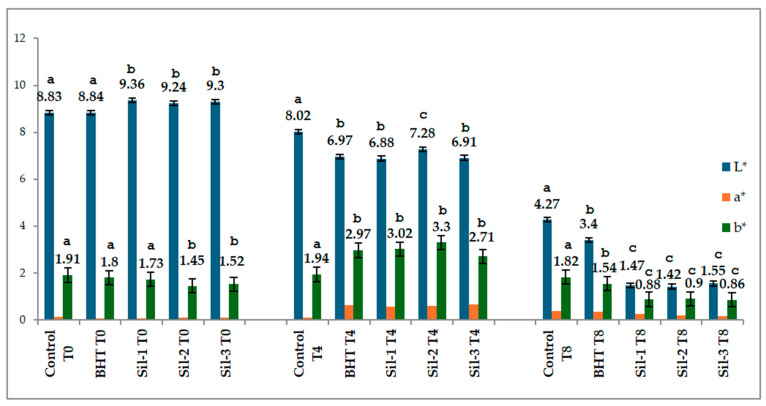
Color parameters (L*, a*, b*). Data for each group of color parameters with different superscript letters are significantly different (*p* < 0.05).

Colorimetric evaluation (Figure 6) showed that SIL-O supplementation did not negatively affect the initial appearance of sunflower oil, as reflected by L* values comparable to or slightly higher than those of the control. During thermal treatment, all samples exhibited progressive color changes, with a marked decrease in L* and dynamic variations in a* and b*, indicating the development of thermo-oxidative degradation.

The transient increase in a* and b* values at T4 may reflect intermediate stages of pigment transformation and secondary oxidation product formation. Such behavior is consistent with literature reports showing that heating of edible oils leads to darkening, red-shift, and alterations in yellow coloration as oxidation progresses [41,42].

At T8, the b* values in the SIL-O samples decreased to 0.86–0.90, below those of the control (1.82) and BHT (1.54), indicating a reduction in yellow tone as degradation progressed. This type of response can be interpreted as the effect of the consumption or transformation of the initial colored compounds and the appearance of new oxidation products. The literature on heated oils shows that changes in b* reflect changes in pigments, secondary oxidation products, and browning compounds, and the direction of the change depends on the matrix, temperature, and the nature of the antioxidant [42].

### 2.5. Correlation Between Parameters

#### 2.5.1. Pearson Correlation Between Parameters

**Figure 7:**
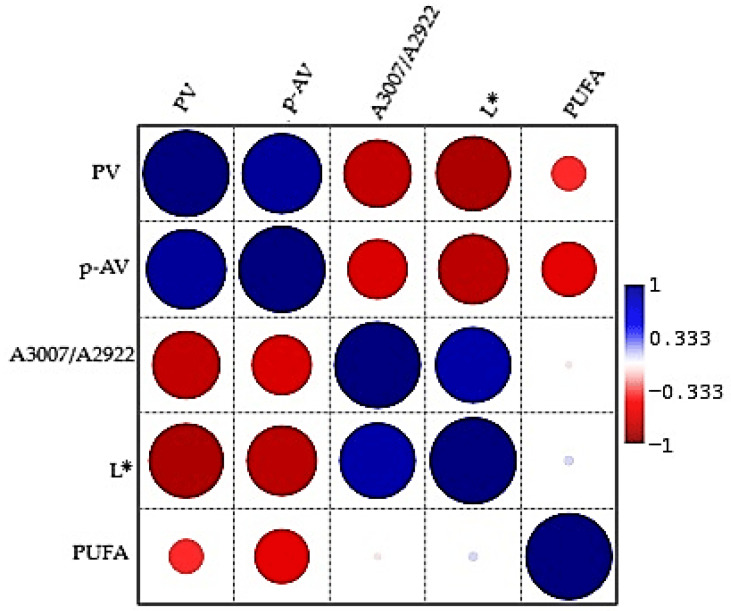
Pearson correlation heatmap of oxidation, fatty acids and spectral indices of oil samples treated with SIL-O and subjected to heating. The size and color of the circles represent the strength and direction of Pearson correlation coefficients (r), respectively. Larger circles indicate stronger correlations (higher absolute values of r), while smaller circles correspond to weaker associations. The color scale reflects the sign of the correlation: blue denotes positive correlations (r > 0), whereas red indicates negative correlations (r < 0). The intensity of the color is proportional to the magnitude of the correlation coefficient.

To better understand the relationships between oxidative stability parameters and fatty acid composition, a correlation matrix was constructed (Figure 7). The correlation analysis was refined to focus on relationships that provide meaningful insight into the oxidation process, excluding mathematically inherent associations.

A very strong positive correlation was observed between the peroxide value (PV) and the p-anisidine value (p-AV), indicating that the primary and secondary oxidation processes occur simultaneously and are closely interconnected. Both PV and p-AV showed strong negative correlations with the FTIR spectral ratio (A3007/A2922) and the brightness parameter (L*), suggesting that increased oxidation is associated with the degradation of unsaturated bonds and a decrease in the brightness of the sample.

The A3007/A2922 ratio showed a strong positive correlation with L*, indicating that this spectral parameter is closely related to lipid structural integrity and optical properties. This suggests that higher levels of unsaturation are associated with higher brightness values, reflecting better preservation of the lipid matrix.

Polyunsaturated fatty acids (PUFA) showed negative correlations with both PV and p-AV, confirming that polyunsaturated fractions are preferentially degraded during oxidative processes. In contrast, PUFA showed weak positive correlations with A3007/A2922 and L*, further supporting their association with lipid quality and structural stability.

Overall, the correlation analysis demonstrates a clear inverse relationship between lipid oxidation and structural integrity. While PV and p-AV characterize the progression of oxidative degradation, A3007/A2922, L* and PUFA reflect the preservation of lipid quality.

#### 2.5.2. Principal Component Analysis (PCA)

**Figure 8:**
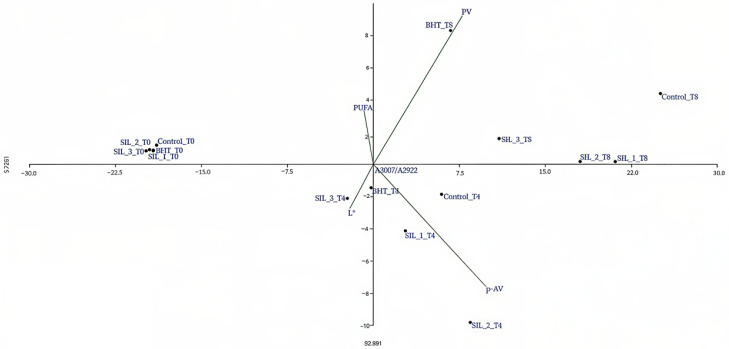
PCA correlation matrix of oxidation of oil samples treated with SIL-O and subjected to heating.

Principal Component Analysis (PCA) was performed in order to evaluate the influence of storage time and antioxidant treatments on the oxidative stability and fatty acid profile of the analyzed oil samples (Figure 8). The PCA results revealed a clear differentiation of samples according to both thermal process duration (T0, T4, T8) and treatment type (Control, BHT, SIL_1, SIL_2, SIL_3), highlighting their impact on lipid oxidation processes.

The first principal component (PC1) explained 92.89% of the total variance, while the second principal component (PC2) accounted for 5.73%, indicating that most of the variability in the data set is determined by oxidation-related processes.

PC1 was mainly associated with oxidation markers, especially the peroxide value (PV), the p-anisidine value (p-AV) and the FTIR spectral ratio (A3007/A2922). PC2 contributed to the differentiation of samples according to the type of oxidation products, separating primary oxidation (associated with PV) from secondary oxidation (associated with p-AV).

At the initial stage (T0), all samples (Control_T0, BHT_T0, SIL_T0 variants) were clustered on the negative side of PC1, indicating minimal oxidation and similar initial quality in all treatments. This confirms that no significant oxidative degradation occurred before storage.

After 4 days of storage (T4), the samples showed increased dispersion. The T4 control samples moved towards the positive side of PC1, indicating the onset of oxidation, while the SIL-treated samples showed a variable behavior. SIL_1_T4 and SIL_2_T4 were positioned in the lower quadrant (negative PC2), suggesting a higher contribution of secondary oxidation products (p-AV), while BHT_T4 remained closer to the origin, indicating a better protective effect against oxidation.

Upon extended storage (T8), a clear separation of samples along PC1 was observed. The T8 control samples were positioned furthest on the positive side of PC1, indicating the highest level of oxidation. The SIL-treated samples (especially SIL_1_T8 and SIL_2_T8) also shifted towards positive values of PC1, but remained closer to the origin compared to the control, suggesting a delay in the progression of oxidation. The BHT_T8 samples were associated with PV and positioned along the positive direction of PC2, indicating the predominance of primary oxidation products.

The orientation of the variable vectors further supports these observations. The PV vector is directed towards the upper quadrant, while the p-AV vector is directed towards the lower quadrant, confirming the distinction between primary and secondary oxidation processes. The ratio A3007/A2922 is aligned with PC1, indicating a strong relationship with the overall progression of oxidation.

Overall, the PCA results demonstrate that time of thermal treatment is the dominant factor influencing lipid oxidation, while antioxidant treatments modulate both the rate and type of oxidation. SIL-derived treatments showed a partial protective effect by delaying oxidation compared to the control group, although their effectiveness varied depending on the concentration and time of thermal process. These findings are consistent with the Pearson correlation results, confirming that oxidation processes are closely associated with the depletion of unsaturated fatty acids and the deterioration of physicochemical properties.

## 3. Materials and Methods

### 3.1. Obtaining of SIL-O Derivative

The esterified derivative of silibinin (SIL-O) was obtained by in-house green synthesis as is presented in our previous study [7].

The materials used are: Silibinin, analytical grade SIL (HPLC, ≥98%), Oleic acid (OA) analytical grade (GC, ≥99%), Acetone, anhydrous, pre-dried over 4 Å molecular sieves, (HPLC, ≥99.9%), Methanol, analytical grade, (AR, ≥99.5%), Chloroform, analytical grade, (HPLC,/GC, ≥99.9%), Ethyl acetate, analytical grade. All chemicals were purchased from Sigma Aldrich, Merck KGaA (Darmstadt, Germany).

For the green synthesis, lipase-catalyzed esterification was performed, using Novozyme 435 (immobilized lipase, 10,000 U/g), procured from Novonesis (Kongens Lyngby, Denmark). The reference antioxidant BHT (Butylated Hydroxytoluene, purity ≥99%) as positive control was purchased from Sigma-Aldrich (Steincheim am Albuch, Germany).

Refined, deodorized sunflower oil (single batch) was used as the lipid matrix. Prior to experimentation, the oil was stored in amber glass containers at room temperature, protected from light.

**Figure 9:**
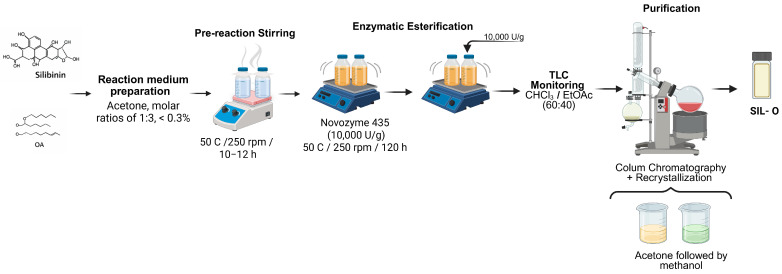
Flow process for synthesis of SIL-O compound.

The reaction medium was prepared by dissolving silibinin with oleic acid in anhydrous acetone dried over 4 Å molecular sieves. The substrates were used in a molar ratio of 1:3, while the water content was maintained below 0.3% to ensure optimal enzymatic activity. The reaction mixture was transferred into sealed glass vessels and magnetically stirred at 250 rpm and 50 °C for 10–12 h to achieve complete solubilization and pre-equilibration. Enzymatic esterification was initiated by adding Novozyme^®^ 435 (immobilized lipase, 10,000 U/g), followed by incubation on an orbital shaker (250 rpm, 50 °C) for 120 h. Reaction progress was monitored by thin-layer chromatography (TLC) on silica gel plates (Merck KGaA, Darmstadt, Germany) using chloroform/ethyl acetate (60:40, *v*/*v*) as the mobile phase. After completion, the reaction mixtures were purified by column chromatography to remove unreacted substrates and byproducts. The collected fractions containing the target esters were concentrated under reduced pressure and further purified by recrystallization (acetone followed by methanol), yielding high-purity silibinin derivatives suitable for further analyses. The process is presented in Figure 9.

### 3.2. Preparation of Antioxidant-Enriched Oils

SIL-O was incorporated into sunflower oil at predefined concentrations to obtain a dose–response evaluation. Briefly, the oil was gently warmed to 35–40 °C to reduce viscosity and facilitate mixing. SIL-O was pre-dissolved in a minimal volume of food-compatible volatile solvent (ethanol or isopropanol) and then added to the oil under continuous magnetic stirring for 15–20 min. The solvent was subsequently removed at 40 °C under a mild nitrogen stream to ensure comparable residual solvent levels across all samples. The control oil underwent the same procedure with the same solvent volume but without antioxidant addition. A BHT-supplemented oil was prepared analogously. All prepared oils were transferred to amber glass bottles and equilibrated for 12–24 h prior to thermal treatment.

Experimental groups. Oils were evaluated as: (i) control (no antioxidant), (ii) SIL-O-enriched oils (100, 200, and 300 mg/kg), and (iii) BHT (200 mg/kg). Each group was prepared in triplicate as independent batches.

The selection of the SIL-O concentration range (100–300 ppm) was guided by both technological relevance and regulatory benchmarks, particularly in relation to the commonly used synthetic antioxidant butylated hydroxytoluene (BHT). In food applications, BHT is typically employed at concentrations around 200 ppm, which is widely recognized as an effective and safe level for oxidative stabilization of lipid systems. Therefore, in order to enable a meaningful comparison, we selected one concentration below (100 ppm), one equivalent (200 ppm), and one above (300 ppm) this reference value.

After antioxidant addition, the oil samples were stored for approximately 3–4 days prior to GC–MS analysis under controlled conditions (darkness, limited oxygen exposure, and ambient temperature). Therefore, the samples labeled as T0 represent a post-addition baseline rather than an immediate zero-time measurement.

### 3.3. Thermal Oxidation Model at 180 °C and Sampling Strategy

To evaluate antioxidant performance under conditions relevant to culinary heating, the oils were subjected to controlled thermal oxidation. For each replicate, a fixed mass of oil (100 g) was placed in identical open borosilicate glass vessels to standardize the oil–air interface. Samples were heated at 180 ± 2 °C using a temperature-controlled heating system (Esmach, Esmach Ali Group S.r.l., Grisignano, Italy) and temperature was continuously monitored. No food matrix was introduced to minimize variability and to isolate oxidative changes attributable to thermal stress and antioxidant supplementation.

Aliquots were collected at baseline (0 h) and after 4 h and 8 h of heating (Table 3).

### 3.4. Determination of Oxidation Parameters

#### 3.4.1. Determination of Peroxide Value (PV) and Inhibition of Oil Oxidation (IO)

Peroxide value (PV) was determined by iodometric titration, according to a standard method for primary oxidation products (hydroperoxides) [43]. Briefly, an accurately weighed lipid sample (5 g) was dissolved in 30 mL acetic acid–chloroform mixture (3:2, *v*/*v*), followed by the addition of 0.5 mL saturated potassium iodide. After incubation in the dark, the liberated iodine was titrated with standardized sodium thiosulfate using starch as indicator. PV was expressed as milliequivalents of active oxygen per kilogram of fat (meq O_2_/kg), calculated as:

(1) $$PV=\frac{\left(Vs-Vb\right)\times N\times 1000}{m}$$

where Vs is the titrant volume for the sample (mL), Vb is the blank volume (mL), N is the normality of sodium thiosulfate, and m is the sample mass (g).

#### 3.4.2. Determination of p-Anisidine Value (p-AV)

The p-anisidine value (p-AV), reflecting secondary oxidation products (mainly aldehydes), was determined spectrophotometrically [44]. The lipid sample was dissolved in iso-octane and the absorbance was measured at 350 nm before and after reaction with p-anisidine reagent. The p-AV was calculated using:

(2) $$p-AV=\frac{25\;(1.2\;A2-A1)}{m}$$

where A1 is the absorbance of the oil solution at 350 nm before reagent addition, A2 is the absorbance after reaction with p-anisidine, and m is the mass of sample (g) used to prepare the solution.

#### 3.4.3. Determination of Total Oxidation Value (TOTOX)

The total oxidation value (TOTOX) was calculated as an integrated indicator of primary and secondary oxidation:

(3) TOTOX=2×PV+p-AV

where PV—peroxide value and p-AV—p-Anisidine Value determined as above.

### 3.5. ATR-FTIR Spectroscopy

ATR-FTIR spectra were recorded to monitor molecular changes associated with thermal oxidation. Measurements were performed using an ATR-FTIR spectrometer (Nicolet Is50 FT-IR, Thermo Fisher Scientific, Waltham, MA, USA). Spectra were acquired in the range 4000–650 cm^−1^ at a resolution of 4 cm^−1^ using 32 scans per spectrum. Before each measurement series, a background spectrum was collected. For each sample, a small amount of oil was placed directly onto the ATR crystal, and spectra were recorded at controlled ambient temperature (20–25 °C). The crystal was thoroughly cleaned with isopropanol between measurements to prevent cross-contamination [3].

Spectral data were baseline-corrected and normalized (e.g., vector normalization or normalization to the aliphatic stretching region) to ensure comparability among samples. Oxidation-related spectral features were evaluated through changes in characteristic bands, particularly those associated with unsaturation and the formation of oxidation products: the cis double bond stretching band (~3007 cm^−1^), the ester carbonyl band (~1743 cm^−1^), the carbonyl region attributable to secondary oxidation products (~1710–1725 cm^−1^), and the trans/conjugated system region (~965 cm^−1^). In addition, unsaturation index (A3007/A2922) was calculated to summarize oxidative progression,

### 3.6. Fatty Acid Composition by GC-MS

#### 3.6.1. Fatty Acid Methyl Ester (FAME) Preparation

All solvents and reagents used for fatty acid derivatization and GC–MS analysis were of chromatographic grade and were purchased from Sigma-Aldrich (St. Louis, MO, USA). Fatty acids were converted to their corresponding methyl esters prior to chromatographic analysis. Briefly, 100 µL of oil sample was placed in a 15 mL glass vial and mixed with 5 mL of methanol. Subsequently, 100 µL of acetyl chloride was added dropwise under continuous stirring. The reaction mixture was allowed to proceed for 45 min at room temperature, protected from light to minimize oxidative degradation. The methylation reaction was quenched by the addition of 3 mL of 0.25 M potassium carbonate solution. Fatty acid methyl esters were then extracted by adding 1 mL of hexane and stirring the mixture for 1 h in the dark. The organic phase was collected, dried under a gentle stream of nitrogen, and the residue was re-dissolved in 100 µL of hexane. An aliquot of 1 µL was injected into the GC–MS system for analysis [45].

#### 3.6.2. GC-MS Analysis Protocol

FAME analysis was performed using an HP6890 gas chromatograph coupled to an HP5973 mass selective detector (Agilent Technologies, Palo Alto, CA, USA). Samples (1 µL) were injected in splitless mode and separated on a VF-5MS capillary column (5% phenyl-arylene–95% dimethylpolysiloxane; 30 m × 0.25 mm i.d.; 0.25 µm film thickness (Varian Inc., Palo Alto, CA, USA). The oven temperature program was set from 50 °C to 230 °C at a rate of 4 °C/min, followed by a final hold of 5 min. Helium was used as the carrier gas at a constant flow rate of 1 mL/min. The MS ion source temperature was set at 250 °C, with an ionization energy of 70 eV and a mass scan range of 50–600 amu. A solvent delay of 3 min was applied [45].

Fatty acid methyl esters were identified by comparison of their mass spectra with those from the NIST11 library, accepting matches with a similarity index greater than 95%, using ChemStation software (version B.01.00). The results were expressed as relative area percentages by normalizing individual peak areas to the total FAME area. Under these conditions, minor analytical variations may be amplified, particularly for fatty acid classes present at low concentrations. Solvent blanks were analyzed under identical conditions to verify the absence of contamination.

#### 3.6.3. Fatty Acid Main Classes Calculation

The fatty acid analysis focused on the major components of sunflower oil. SFA were represented by palmitic (C16:0) and stearic acid (C18:0), MUFA by oleic acid (C18:1), and PUFA by linoleic acid (C18:2). Although minor fatty acids were not included, these four compounds constitute the predominant fraction of the lipid profile and are widely used as representative markers for evaluating oxidation-related changes in vegetable oils. Relative changes in fatty acid classes during thermal treatment were calculated with the formulas:

Δ (SFA, MUFA, PUFA) = [*Area*% (SFA, MUFA, PUFA) *tx* − *Area*%(SFA, MUFA, PUFA) *to*] × 100 (4)

where *Area%tx* represents the relative percentage of the fatty acid (or fatty acid class) at the given sampling time (T4 or T8), and *Area%to* represents the corresponding relative percentage at the initial time point (T0), used as baseline.

### 3.7. Color Analysis

The color attributes of samples were evaluated using a colorimeter (Konica Minolta, Tokyo, Japan), according to the CIE L*a*b* color system. Within this framework, the parameter L* quantifies lightness on a scale from 0 (black) to 100 (white), a* represents the green (−) to red (+) chromatic axis, while b* describes the blue (−) to yellow (+) axis [46].

### 3.8. Statistical Analysis

Data were reported as mean ± standard deviation. Differences among treatments and heating times (0, 4, and 8 h) were evaluated using analysis of variance (two-way ANOVA: treatment × time), followed by post hoc test (Tukey) for multiple comparisons. Statistical significance was set at *p* < 0.05. When assumptions of normality or homoscedasticity were not met, nonparametric alternatives were applied. Principal Component Analysis (PCA) and Pearson correlation analysis were performed using PAST software (version 4.03) on standardized data to assess relationships between oxidative stability parameters and fatty acid composition.

## 4. Conclusions

The present study demonstrates that silibinin oleate (SIL-O), as a lipophilic derivative of a natural polyphenol, can contribute to limiting thermo-oxidative degradation in sunflower oil, with a concentration-dependent effect. To synthesize the main results:

- Thermal treatment significantly increased PV, p-anisidine, and TOTOX, indicating progressive oxidation.
- FTIR analysis confirmed molecular changes associated with the loss of unsaturation and formation of oxidation products.
- Fatty acid evolution showed preferential degradation of PUFA, consistent with typical oxidation mechanisms in sunflower oil.
- Antioxidant addition improved oxidative stability, with BHT showing the highest efficiency at equal concentration and SIL-O exhibiting a comparable, dose-dependent effect.
- Correlation analysis revealed consistent relationships between oxidation indices, PUFA content, FTIR unsaturation index, and color parameters, supporting a coherent link between chemical degradation, molecular structural changes, and visual quality evolution.

Direct comparison with BHT is valid only at equivalent concentrations, while improved performance at higher SIL-O levels reflects a dose-dependent response. The thermal behavior of SIL-O under prolonged heating remains to be further clarified.

Within these limitations, the study suggests that lipophilized derivatives of natural antioxidants may represent a promising approach for improving the stability of PUFA-rich oils. The findings support the concept that structural modification of phenolic compounds through lipophilization enhances their compatibility with lipid matrices and their functionality under high-temperature processing conditions.

From an originality perspective, this study does not rely on the novelty of the analytical techniques themselves, but rather on their integrated application to evaluate the behavior of a lipophilic silibinin derivative in a complex lipid system under severe thermal conditions. The results provide insight into the dose-dependent antioxidant performance of SIL-O and its ability to modulate thermo-oxidative degradation in PUFA-rich oils.

The contribution of this study lies in demonstrating that structural modification of a natural polyphenol through lipophilization can enhance its functionality in lipid matrices, offering a viable approach for the development of natural antioxidants adapted to high-temperature processing conditions.

From an originality perspective, the study introduces the use of a silibinin lipophilic derivative directly in a real food model subjected to severe thermal stress, offering new insights into its potential as a functional ingredient in edible oils.

From an application standpoint, SIL-O may be considered a viable candidate for the development of stabilized oils intended for high-temperature uses, while also contributing to the advancement of nutraceutical-oriented formulations.

Future research should focus on the evaluation of long-term stability under industrial conditions, detailed kinetic mechanisms of antioxidant action, interactions with food matrix components, as well as safety, bioavailability, and sensory impact in final products.

## Figures and Tables

**Table 1:** Peroxide value (PV), para-anisidine value (p-AV) and TOTOX values of sunflower oil samples with SIL-O and BHT, before and after 4 and 8 h of heat exposure, compared to the control.

No.	Abbreviation	PV (meq O_2_/kg)	p-AV	TOTOX
1	Control_T0	1.75 ± 0.03 ^a^	4.35 ± 0.32 ^a^	7.85 ^a^
2	SIL_1_T0	1.36 ± 0.02 ^c^	4.41 ± 0.38 ^a^	7.13 ^a^
3	SIL_2_T0	1.33 ± 0.02 ^c^	3.92 ± 0.20 ^b^	6.58 ^c^
4	SIL_3_T0	0.96 ± 0.01 ^d^	3.89 ± 0.17 ^b^	5.81 ^d^
5	BHT_T0	1.42 ± 0.03 ^b^	4.17 ± 0.03 ^b^	7.01 ^b^
6	Control_T4	15.93 ± 0.02 ^a^	24.71 ± 0.43 ^b^	56.58 ^a^
7	SIL_1_T4	11.49 ± 0.03 ^b^	24.00 ± 0.04 ^b^	46.98 ^b^
8	SIL_2_T4	10.63 ± 0.01 ^c^	32.02 ± 0.66 ^a^	53.28 ^a^
9	SIL_3_T4	9.78 ± 0.02 ^d^	18.85 ± 0.02 ^c^	38.40 ^c^
10	BHT_T4	11.75 ± 0.01 ^b^	19.97 ± 0.63 ^c^	43.47 ^b^
11	Control_T8	32.03 ± 0.02 ^a^	36.06 ± 0.06 ^a^	93.37 ^a^
12	SIL_1_T8	24.83 ± 0.02 ^b^	36.24 ± 1.44 ^a^	83.38 ^b^
13	SIL_2_T8	22.94 ± 0.49 ^c^	33.71 ± 0.29 ^b^	73.26 ^c^
14	SIL_3_T8	19.48 ± 0.47 ^d^	27.37 ± 0.25 ^d^	38.96 ^d^
15	BHT_T8	22.96 ± 0.02 ^c^	29.31 ± 0.06 ^c^	82.16 ^b^

Values are presented as means ± standard deviation (n = 3). Data in the same column, for each group of treatment (initial, after 4 and 8 h of heating at 180 °C) with different superscript letters are significantly different (*p* < 0.05).

**Table 2:** FTIR unsaturation index (A3007/A2922) of oil samples.

No.	Abbreviation	A1743	A3007	A2922	Unsaturation Index A3007/A2922
1	Control_T0	57.626	96.063	65.175	1.474
2	SIL_1_T0	57.666	96.038	65.166	1.474
3	SIL_2_T0	57.621	96.011	65.115	1.474
4	SIL_3_T0	57.573	95.921	65.026	1.475
5	BHT_T0	57.750	96.067	65.210	1.473
6	Contol_T4	57.734	96.346	65.129	1.479
7	SIL_1_T4	57.774	96.413	65.101	1.481
8	SIL_2_T4	58.026	96.780	65.214	1.484
9	SIL_3_T4	58.045	96.779	65.181	1.485
10	BHT_T4	57.619	95.987	64.864	1.480
11	Control_T8	57.897	65.143	96.616	0.674
12	SIL_1_T8	58.045	65.199	97.118	0.671
13	SIL_2_T8	58.143	65.243	97.139	0.672
14	SIL_3_T8	58.134	65.241	97.114	0.672
15	BHT_T8	89.211	98.398	72.861	1.350

**Table 3:** The experimental samples and the treatment applied.

No.	Abbreviation	Composition	Heating Treatment
1	Control_T0	Sunflower oil (SF) without addition	No heating
2	SIL_1_T0	Sunflower oil (SF) + 100 ppm silibinin oleate (SO)	No heating
3	SIL_2_T0	Sunflower oil (SF) + 200 ppm silibinin oleate (SO)	No heating
4	SIL_3_T0	Sunflower oil (SF) + 300 ppm silibinin oleate (SO)	No heating
5	BHT_T0	Sunflower oil (SF) + 200 ppm BHT	No heating
6	Contol_T4	Sunflower oil (SF)	4 h at 180 °C
7	SIL_1_T4	Sunflower oil (SF) + 100 ppm silibinin oleate (SO)	4 h at 180 °C
8	SIL_2_T4	Sunflower oil (SF) + 200 ppm silibinin oleate (SO)	4 h at 180 °C
9	SIL_3_T4	Sunflower oil (SF) + 300 ppm silibinin oleate (SO)	4 h at 180 °C
10	BHT_T4	Sunflower oil (SF) + 200 ppm BHT	4 h at 180 °C
11	Control_T8	Sunflower oil (SF)	8 h at 180 °C
12	SIL_1_T8	Sunflower oil (SF) + 100 ppm silibinin oleate (SO)	8 h at 180 °C
13	SIL_2_T8	Sunflower oil (SF) + 200 ppm silibinin oleate (SO)	8 h at 180 °C
14	SIL_3_T8	Sunflower oil (SF) + 300 ppm silibinin oleate (SO)	8 h at 180 °C
15	BHT_T8	Sunflower oil (SF) + 200 ppm BHT	8 h at 180 °C

## Data Availability

The original contributions presented in the study are included in the article; further inquiries can be directed to the corresponding author.
